# Impact of Left Atrial Appendage Morphology on Recurrence in Embolic Stroke of Undetermined Source and Atrial Cardiopathy

**DOI:** 10.3389/fneur.2021.679320

**Published:** 2021-06-22

**Authors:** Dong-Seok Gwak, WooChan Choi, Yong-Won Kim, Yong-Sun Kim, Yang-Ha Hwang

**Affiliations:** ^1^Department of Neurology, Kyungpook National University Hospital, Daegu, South Korea; ^2^Department of Neurology, School of Medicine, Kyungpook National University, Daegu, South Korea; ^3^Department of Radiology, Kyungpook National University Hospital, Daegu, South Korea; ^4^Department of Radiology, School of Medicine, Kyungpook National University, Daegu, South Korea

**Keywords:** recurrence, embolic strokes, atrial appendage, cerebral infarction, CT scan

## Abstract

**Background:** The left atrial appendage (LAA) is a major source of thrombus and non-chicken wing (CW). LAA morphology is a risk factor for embolic events in atrial fibrillation. However, the association of non-CW morphology with embolic stroke recurrence is unknown in patients with embolic stroke of undetermined source (ESUS) and atrial cardiopathy.

**Methods:** We conducted retrospective analyses using a prospective institutional stroke registry (2013–2017). Patients with ESUS and atrial cardiopathy were enrolled. Atrial cardiopathy was diagnosed if an increased left atrial diameter (>40 mm, men; >38 mm, women), supraventricular tachycardia, or LAA filling defect on computed tomography (CT) were present. Patients admitted >24 h after onset were excluded. LAA morphology was evaluated using CT and categorized into CW vs. non-CW types. The primary outcome was embolic stroke recurrence. Multivariable Cox proportional hazards models were used to examine the independent association between LAA morphology and outcome.

**Results:** Of 157 patients, 81 (51.6%) had CW LAA morphology. The median follow-up was 41.5 (interquartile range 12.3–58.5) months corresponding to 509.8 patient years. In total, 18 participants experienced embolic stroke recurrences (3.80 per 100 patient-years). Non-CW morphology was more associated with embolic stroke recurrence than CW morphology (hazard ratio (HR), 3.17; 95% confidence interval (CI), 1.13–8.91; *p* = 0.029). After adjusting for CHA_2_DS_2_-VASc score and number of potential embolic sources, non-CW morphology showed an independent association with outcome (adjusted HR, 2.90; 95% CI, 1.02–8.23; *p* = 0.045).

**Conclusions:** The LAA morphology types may help identify high risk of embolic stroke recurrence in ESUS with atrial cardiopathy. LAA morphology in atrial cardiopathy may provide clues for developing therapies tailored to specific mechanisms.

## Introduction

The concept of embolic stroke of undetermined source (ESUS) assumed that most cryptogenic strokes are thromboembolic and could benefit from anticoagulation (1). However, the failure of the two recent clinical trials to demonstrate a reduction in stroke recurrence with anticoagulants for patients with ESUS facilitates the conceptual change that ESUS comprises heterogeneous subgroups (2, 3). One of the subgroups of ESUS is atrial cardiopathy, which leaves atrial substrates with structural, functional, or electrical remodeling preceding clinical atrial fibrillation (AF) (4). Abnormal atrial substrates *per se* could be thrombogenic even in the absence of AF (5–9). Several markers of atrial cardiopathy are associated with subsequent stroke events (10, 11). Moreover, anticoagulant therapy may be beneficial for a subset of these patients with high-risk characteristics for thromboembolism including an enlarged left atrium (12) or elevated N-terminal pro-B-type natriuretic peptides (13).

Another potential biomarker of embolic risk in atrial cardiopathy is the left atrial appendage (LAA) morphology. LAA varies significantly in shape and has usually been categorized into four distinct morphologies: chicken wing (CW), windsock, cactus, and cauliflower (14–16). LAA is a major source of thrombus in patients with AF (17), and, compared with CW morphology, non-CW LAA morphology is related to embolic events (14, 18). However, the association of non-CW morphology with recurrent embolic stroke is unknown in patients with ESUS and atrial cardiopathy. Thus, this study aimed to determine whether non-CW morphology increases the recurrence risk of embolic stroke in this patient population.

## Materials and Methods

### Study Population

**Figure 1:**
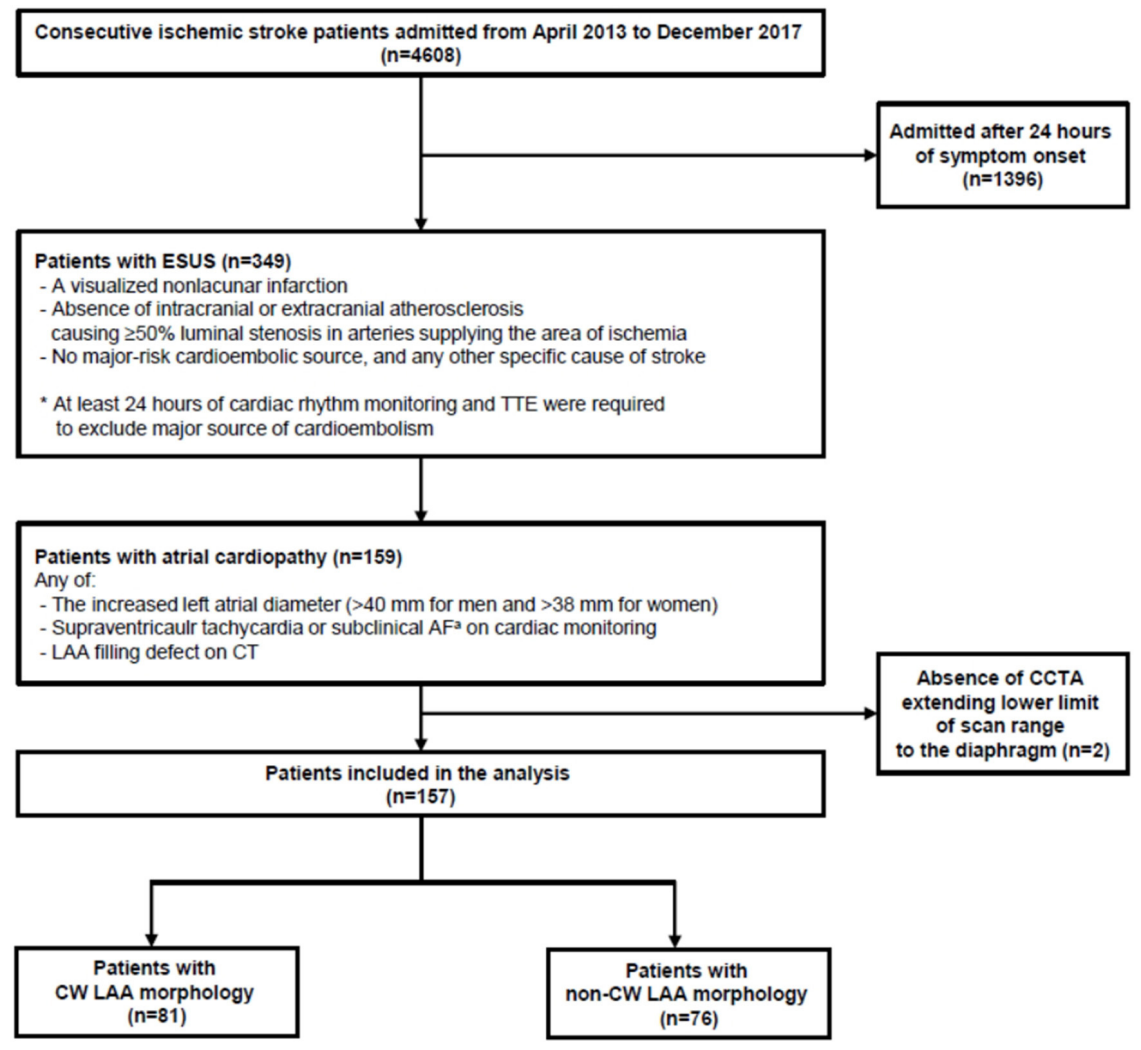
Flowchart of patient screening and enrollment. ESUS, embolic stroke of undetermined source; TTE, transthoracic echocardiography; AF, atrial fibrillation; LAA, left atrial appendage; CT, computed tomography. ^a^Defined as atrial fibrillation for <6 min.

Consecutive stroke patients were retrospectively reviewed from the prospective institutional stroke registry database. The inclusion criteria for this study are as follows: (1) admission to Kyungpook National University Hospital from April 2013 to December 2017, (2) ESUS defined by the Cryptogenic Stroke/ESUS International Working Group (1), and (3) atrial cardiopathy. This is defined as the increased left atrial diameter (>40 mm for men and >38 mm for women), supraventricular tachycardia/subclinical AF, or an LAA filling defect on cerebral computed tomography angiography (CCTA) (19–23). However, patients who were admitted >24 h of symptom onset and who did not have a CCTA extending to the lower limit of the scan range to the diaphragm were excluded. Finally, 157 patients were eligible for analysis (Figure 1). The study protocol was approved by the local institutional review board (IRB approval number: KNUH 2020-10-018). Written informed consent was waived because of the retrospective nature of the study and the anonymity of study subjects.

### Clinical Data Collection

Baseline demographics for study participants were extracted from the institutional stroke registry database or electronic medical records. Potential embolic sources, including arterial atherosclerotic disease (presence of any ipsilesional carotid stenosis of <50% or aortic arch atherosclerosis), left ventricular dysfunction (presence of low left ventricular ejection fraction of 30–35% or regional wall motion abnormality reported in the echocardiogram or presence of heart failure history), cardiac valve disease (presence of moderate to severe stenosis or regurgitation of aortic or mitral valve identified in the echocardiogram), patent foramen ovale (any grade; detected on the echocardiogram), and cancer (history of cancer, except skin cancer, at baseline), were collected (20, 21). The CHA_2_DS_2_-VASc score, which is a well-validated tool for stratifying the risk of stroke in patients with AF or ESUS, was calculated (sum of the point values of variables of congestive heart failure, hypertension, age (1 point, 65–74; 2 points, ≥75), diabetes mellitus, prior stroke or transient ischemic attack (2 points), vascular disease, and female sex) (24, 25). Moreover, echocardiographic findings, LAA filling defect on CCTA, supraventricular tachycardia (paroxysmal tachyarrhythmia with narrow QRS complex and regular ventricular response which is initiated by atrial or atrioventricular node) (26) or subclinical AF (AF detected during cardiac monitoring lasting <6 min) (20), and AF detection during the follow-up period were collected. Transesophageal echocardiography (TEE) parameters, including LAA emptying velocity (the peak velocity of the flow out of the LAA) (18) and spontaneous echogenic contrast (SEC; visually diagnosed by the presence of characteristic swirling smoke-like echoes distinguished from background white noise) (27) on LAA, were also collected. The primary outcome was embolic stroke recurrence defined as sudden onset focal neurological deficits lasting for >24 h with evidence of acute non-lacunar brain infarct and no relevant arterial stenosis on brain imaging. The secondary outcomes were ischemic stroke recurrence, any stroke (ischemic or hemorrhagic) recurrence, and all-cause mortality.

### Assessment of LAA Morphology

**Figure 2:**
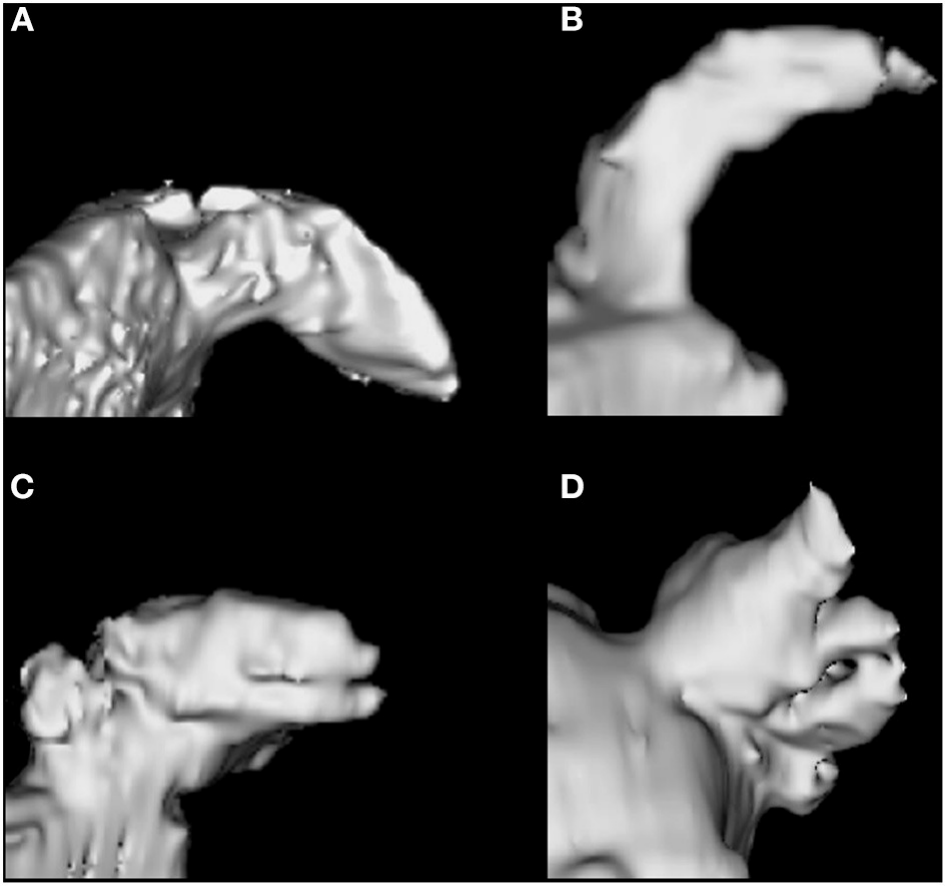
Left atrial appendage morphology types. Chicken wing **(A)**, Windsock **(B)**, Cactus **(C)**, Cauliflower **(D)**.

According to the institutional protocol, CCTA was obtained on admission to the emergency department in patients with suspected acute ischemic stroke within 24 h of symptom onset (The detailed imaging protocol is described in Supplementary Figure 1). The CCTA source images were then post-processed to create three-dimensional volumetric reconstructions of LAA using an open-source program for biomedical research, 3D Slicer (version 4.10.2., http://www.slicer.org). Two vascular neurologists blinded to outcomes (Gwak D-S and Choi WC) visually analyzed LAA morphology according to the previously reported classifications (14–16) and assigned it to two classes (CW vs. non-CW type—windsock, cactus, and cauliflower type; Figure 2). The CW type is characterized by an obvious bend <100° in the proximal or middle part of the dominant lobe; windsock morphology presents one dominant lobe of sufficient length with or without secondary lobes with bending >100°; cactus presents a dominant central lobe with one or more secondary lobes extending from the central lobe; and cauliflower is characterized by the limited overall length with complex internal structures. The discrepancies in LAA morphology were resolved by consensus (Kappa index, 0.770).

### Statistical Analysis Plan

Time-to-event data were summarized by the number of events per patient-years of exposure and examined using the Kaplan–Meier curves, with comparisons between the groups with CW and non-CW morphologies made with the log-rank test. Univariate and multivariable Cox proportional hazards regression analyses were performed to examine the independent association of non-CW LAA morphology with outcomes. Several multivariable models were constructed with prespecified covariates or variables imbalanced between the groups with CW and non-CW morphologies due to the small sample size. Model 1 was adjusted for age, sex, and baseline National Institute of Health Stroke Scale score. Model 2 was adjusted for variables of potential risks for outcomes (CHA_2_DS_2_-VASc score and number of potential embolic sources). Model 3 included the imbalanced baseline characteristics and cardiac biomarkers between the two groups with *p* < 0.10. Associations were reported as hazard ratios (HRs) and 95% confidence intervals (CIs). Those who were available for TEE parameters including LAA emptying velocity and SEC on LAA were subsequently analyzed between the two groups. A two-sided *p* < 0.05 was considered statistically significant. All statistical analyses were performed using SPSS software (version 25.0, IBM SPSS, Armonk, NY, USA). The Kaplan–Meier curves were generated by using GraphPad Prism (version 9.0.0, GraphPad Software, Inc., La Jolla, CA, USA).

## Results

The cohort dataset comprised 349 patients with ESUS. Among them, 157 patients with atrial cardiopathy were included. Of the patients with different LAA morphologies, 81 (51.6%), 33 (21.0%), 25 (15.9%), and 18 (11.5%) were categorized as CW, windsock, cactus, and cauliflower, respectively. The baseline demographics and clinical characteristics were compared between patients with CW and non-CW LAA morphologies (Table 1). The mean age was 69.5 ± 11.6 years with 58% were men. Other variables were not statistically different between the two groups except for hyperlipidemia (43.4 vs. 27.2%, *p* = 0.033). Factors associated with atrial cardiopathy are shown in Table 2. No significant differences in cardiac markers exist between the two groups.

**Table 1:** Baseline demographics and clinical characteristics.

	**All**	**Chicken wing**	**Non-chicken wing**	***P***
	**(*n* = 157)**	**(*n* = 81)**	**(*n* = 76)**	
Age	69.5 ± 11.6	68.7 ± 12.5	70.3 ± 10.6	0.365
Men	91 (58.0)	46 (56.8)	45 (59.2)	0.759
Baseline NIHSS	3.0 (1.0–7.0)	2.0 (1.0–6.5)	3.5 (1.0–7.8)	0.434
Pre-stroke mRS, 0–1 (%)	136 (86.6)	72 (88.9)	64 (84.2)	0.389
Hypertension	96 (61.1)	44 (54.3)	52 (68.4)	0.070
Diabetes mellitus	34 (21.7)	13 (16.0)	21 (27.6)	0.078
Hyperlipidemia	55 (35.0)	22 (27.2)	33 (43.4)	0.033
Coronary artery disease	16 (10.2)	7 (8.6)	9 (11.8)	0.508
Congestive heart failure	8 (5.1)	3 (3.7)	5 (6.6)	0.485
Prior stroke or TIA	17 (10.8)	8 (9.9)	9 (11.8)	0.692
Smoking	40 (25.5)	24 (29.6)	16 (21.1)	0.218
**Potential embolic sources**				
Arterial atherosclerotic disease	103 (65.6)	50 (61.7)	53 (69.7)	0.291
Left ventricular dysfunction	21 (13.4)	9 (11.1)	12 (15.8)	0.389
Cardiac valve disease	7 (4.5)	4 (4.9)	3 (3.9)	1.000
Patent foramen ovale	38 (24.2)	23 (28.4)	15 (19.7)	0.206
Cancer	17 (10.8)	6 (7.4)	11 (14.5)	0.154
Number of potential embolic sources				0.864
0	25 (15.9)	14 (17.3)	11 (14.5)	
1	88 (56.1)	44 (54.3)	44 (57.9)	
≥2	44 (28.0)	23 (28.4)	21 (27.6)	
CHA2DS2-VASc score	4.4 ± 1.5	4.3 ± 1.5	4.6 ± 1.6	0.172
Treatment on discharge				0.604
No antithrombotic	3 (1.9)	1 (1.2)	2 (2.6)	
Antiplatelet (single)	33 (21.0)	17 (21.0)	16 (21.1)	
DAPT	109 (69.4)	56 (69.1)	53 (69.7)	
Oral anticoagulants	9 (5.7)	4 (4.9)	5 (6.6)	
Antiplatelet(s) and oral anticoagulants	3 (1.9)	3 (3.7)	0 (0.0)	

*Variables are presented as mean ± standard deviation or absolute number (proportions)*.

*NIHSS, National Institute of Health Stroke Scale; mRS, modified Rankin Scale; TIA, transient ischemic attack; DAPT, dual antiplatelet therapy*.

**Table 2:** Comparisons of cardiac markers between patients with and without chicken wing left atrial appendage morphology.

	**All**	**Chicken wing**	**Non-chicken wing**	***P***
	**(*n* = 157)**	**(*n* = 81)**	**(*n* = 76)**	
Echocardiographic findings				
LA size (mm)	40.7 ± 5.8	40.4 ± 6.3	41.0 ± 5.2	0.507
LV EF (%)	60.4 ± 5.7	60.3 ± 5.9	60.6 ± 5.4	0.773
LAA filling defect	43 (27.4)	25 (30.9)	18 (23.7)	0.313
Supraventricular tachycardia or subclinical AF	46 (29.3)	19 (23.5)	27 (35.5)	0.097
AF detection during follow-up	14 (8.9)	10 (12.3)	4 (5.3)	0.120

*Variables are presented as mean ± standard deviation or absolute number (proportions)*.

*LA, left atrium; EF, ejection fraction; LAA, left atrial appendage; AF, atrial fibrillation*.

**Figure 3:**
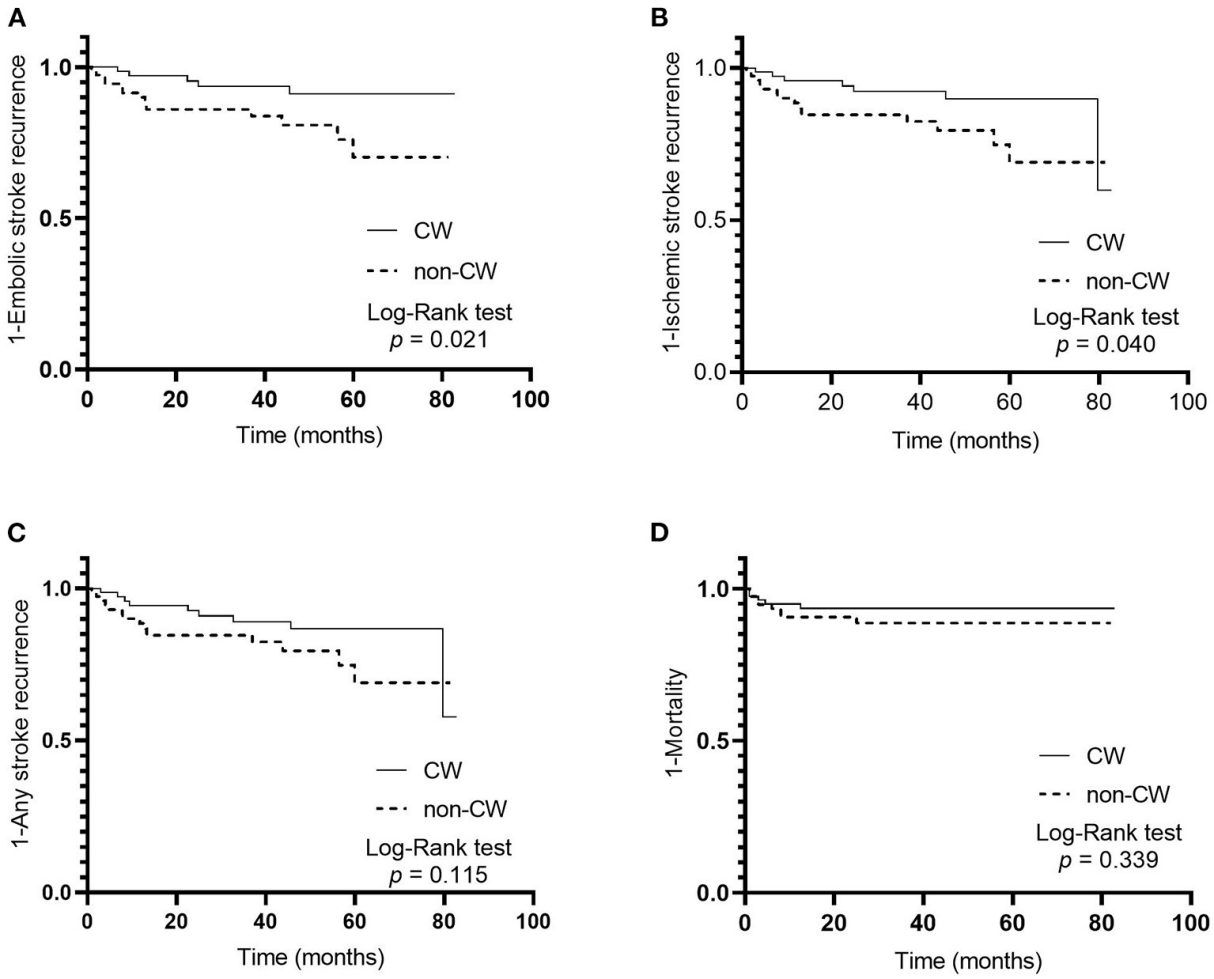
Cumulative probability of primary and secondary outcomes in patients with embolic stroke of undetermined source and atrial cardiopathy according to left atrial appendage morphology; embolic stroke recurrence **(A)**, ischemic stroke recurrence **(B)**, any stroke recurrence **(C)**, and mortality **(D)**.

The median follow-up was 41.5 (interquartile range, 12.3–58.5) months corresponding to 509.8 patient years. In total, 18 (11.5%) embolic stroke recurrences, 21 (13.4%) ischemic stroke recurrences, 23 (14.6%) any stroke recurrences, and 13 (8.3%) deaths corresponded to 3.80, 4.47, 4.90, and 2.55 events per 100 patient years, respectively. The Kaplan–Meier survival curves showed that non-CW morphology was associated with embolic stroke (*p* = 0.021, Figure 3A) and ischemic stroke (*p* = 0.040, Figure 3B) recurrences in patients with ESUS and atrial cardiopathy by log-rank test, respectively. However, the differences in the other secondary outcomes during follow-up were not significant between the two groups (Figures 3C,D).

Non-CW LAA morphology was associated with embolic stroke recurrence in ESUS and atrial cardiopathy (HR, 3.17; 95% CI, 1.13–8.91; *p* = 0.029) in univariate Cox proportional hazards regression analysis. Other variables, including age and CHA_2_DS_2_-VASc score, were also associated with embolic stroke recurrence. Non-CW LAA morphology was independently associated with the recurrence of embolic stroke in multivariable Cox regression models 1–3 (Table 3 and Supplementary Tables 1–3). Age score in model 1 was also independently associated with embolic stroke recurrence. However, no significant independent associations of non-CW morphology exist with secondary outcomes (Table 3 and Supplementary Tables 1–3). TEE parameters were available in 47 patients. The proportion of LAA flow velocity <40 cm/s was similar in patients with non-CW LAA morphology and those with CW morphology (11.8% [2/17] vs. 10.0% [3/30]; *p* = 1.000). However, patients with non-CW LAA morphology showed the presence of SEC in LAA more frequently than those with CW morphology, albeit not statistically significant (52.9% [9/17] vs. 43.3% [13/30]; *p* = 0.526).

**Table 3:** Univariate and multivariable Cox proportional hazards regression analyses investigating associations between non-chicken wing left atrial appendage morphology and outcomes.

**Outcome^**a**^**	**Crude HR**	**Adjusted HR**	**Adjusted HR**	**Adjusted HR**
	**(95% CI)**	**(95% CI)**	**(95% CI)**	**(95% CI)**
		**Model 1^**b**^**	**Model 2^**c**^**	**Model 3^**d**^**
Embolic stroke recurrence	3.17 (1.13–8.91)	3.10 (1.10–8.73)	2.90 (1.02–8.23)	2.95 (1.03–8.43)
Ischemic stroke recurrence	2.52 (1.01–6.29)	2.39 (0.96–5.95)	2.33 (0.93–5.84)	2.31 (0.91–5.83)
Any stroke recurrence	1.95 (0.84–4.52)	1.85 (0.80–4.29)	1.60 (0.70–3.64)	1.58 (0.69–3.63)
Mortality	1.71 (0.56–5.22)	1.56 (0.50–4.91)	1.66 (0.54–5.11)	1.44 (0.44–4.71)

*HR, hazard ratio; CI, confidence interval*.

a *Outcomes are presented as the hazard ratio (95% confidence interval) for values of non-chicken wing compared to chicken wing left atrial appendage morphology*.

b *Model 1 adjusted for age, sex, and baseline NIHSS score*.

c *Model 2 adjusted for CHA_2_DS_2_-VASc score and number of potential embolic sources*.

d *Model 3 adjusted for hypertension, diabetes mellitus, hyperlipidemia, and supraventricular tachycardia or subclinical atrial fibrillation*.

## Discussion

The present study showed that the prevalence of individual LAA morphology types in ESUS and atrial cardiopathy were 51.6% CW and 48.4% non-CW (21% windsock, 15.9% cactus, and 11.5% cauliflower). The event rates of embolic stroke recurrence, ischemic stroke recurrence, any stroke recurrence, and mortality in this population were 3.80, 4.47, 4.90, and 2.55 per 100 patient years, respectively. Furthermore, the risk of embolic stroke recurrence in ischemic stroke patients with ESUS and atrial cardiopathy was independently associated with non-CW LAA morphology, though the associations of non-CW LAA morphology with the recurrence of any ischemic stroke, hemorrhagic stroke, or mortality were not statistically significant.

Several studies regarding LAA morphology and thromboembolism in AF have shown an association of non-CW morphology with an embolic event (14, 16, 18). This could possibly be explained by complex LAA morphology, characterized by extensive LAA trabeculations or increased number of lobes that are more suitable for non-CW than CW morphology, which was more likely to induce blood stasis leading to thrombus formation (16, 28). Furthermore, complex LAA morphology was related to significant LAA dysfunction, including a high degree of SEC (28) and low LAA emptying velocity (29). However, this association has seldom been investigated in the ESUS patient population with atrial cardiopathy.

The data in this study indicated that non-CW LAA morphology in patients with ESUS and atrial cardiopathy may also be associated with thrombus formation leading to the recurrence of embolic stroke. Non-CW LAA morphology tended to have more severe LAA dysfunction than CW morphology, although direct relationships could not be demonstrated due to a small number of patients whose TEE findings were available.

Identifying the factors associated with the high-risk of embolic stroke recurrence in patients with ESUS may aid secondary stroke prevention in clinical practice. Thus, patients with high-risk LAA morphology and ESUS with atrial cardiopathy may respond to anticoagulant rather than antiplatelet agents given the parallel results of the impact of non-CW LAA morphology on stroke events in ESUS with atrial cardiopathy and AF. Further studies are needed to prove the hypothesis.

This study has several limitations. Although several prespecified cox proportional hazards models were tested, the results may not be reliably examined due to the small sample size and limited number of outcome events. Selection bias and unregistered confounding factors cannot be ruled out due to its retrospective study design. Additionally, other potential biomarkers for atrial cardiopathy such as atrial fibrosis (6), P-wave terminal force in lead V1 on ECG (5), and N-terminal pro-brain natriuretic peptide (30) were not analyzed because of lacking data; therefore, interactions and degree of overlap among these biomarkers could not be evaluated. Moreover, the underlying pathophysiologic mechanisms of association between non-CW LAA morphology and the subsequent embolic event cannot be demonstrated because LAA dysfunctions including LAA flow velocity and SEC were evaluated in only a small subgroup of the study participants. Furthermore, the AF detection rate during the median follow-up period of 3.5 years was only 8.9% although all study participants underwent cardiac monitoring over 24 h. The patients with paroxysmal AF or late-onset AF may be underestimated in this study considering the relatively low detection rate compared with other studies (31, 32). Therefore, the association of high-risk LAA morphology with later detection of AF after an index stroke event could not be investigated.

In conclusion, the LAA morphology types may help identify the high risk of embolic stroke recurrence in ESUS with atrial cardiopathy. This biomarker in atrial cardiopathy may provide clues for selecting patients who may benefit from anticoagulants and developing therapies tailored to specific mechanisms.

## Data Availability Statement

The raw data supporting the conclusions of this article will be made available by the authors, without undue reservation.

## Ethics Statement

The studies involving human participants were reviewed and approved by Kyungpook National University Hospital. Written informed consent for participation was not required for this study in accordance with the national legislation and the institutional requirements.

## Author Contributions

D-SG established the study protocol, analyzed and interpreted the data, and wrote the manuscript. WCC, Y-WK, and Y-SK analyzed the data. Y-HH established the study idea, interpreted the data, drafted the manuscript, and made critical revisions in the manuscript with intellectual input.

## Conflict of Interest

The authors declare that the research was conducted in the absence of any commercial or financial relationships that could be construed as a potential conflict of interest.
