# Supplementary material for: Impact of Left Atrial Appendage Morphology on Recurrence in Embolic Stroke of Undetermined Source and Atrial Cardiopathy
Source: Front Neurol. 2021 Jun 22;12:679320. doi: 10.3389/fneur.2021.679320 (PMC8258144; doi:10.3389/fneur.2021.679320)
Supplement: Supplementary file 1 [file Data_Sheet_1.pdf]

Supplementary Table 1.Multivariable Cox proportional hazards regression analysis investigating the association between variables and outcomes (model 1).

| Variables | Embolic stroke recurrence aHR (95% CI) | Ischemic stroke recurrence aHR (95% CI) | Any stroke recurrence aHR (95% CI) | Mortality aHR (95% CI) |
|---|---|---|---|---|
| LAA morphology | 3.10 (1.10–8.73) | 2.39 (0.96–5.95) | 1.85 (0.80–4.29) | 1.57 (0.50–4.92) |
| Age | 1.07 (1.01–1.13) | 1.06 (1.003–1.11) | 1.06 (1.01–1.12) | 1.03 (0.97–1.10) |
| Males | 0.84 (0.31–2.26) | 1.05 (0.42–2.64) | 1.10 (0.45–2.66) | 1.07 (0.33–3.47) |
| Baseline NIHSS score | 1.03 (0.95–1.12) | 1.03 (0.95–1.11) | 1.02 (0.94–1.10) | 1.18 (1.10–1.26) |

Outcomes are presented as the hazard ratio (95% confidence interval) for values of non-chicken wing compared to chicken wing left atrial appendage morphology.

aHR, adjusted hazard ratio; LAA, left atrial appendage; NIHSS, National Institute of Health Stroke Scale

Supplementary Table 2. Multivariable Cox proportional hazards regression analysis investigating the association between variables and outcomes (model 2).

| Variables | Embolic stroke recurrence aHR (95% CI) | Ischemic stroke recurrence aHR (95% CI) | Any stroke recurrence aHR (95% CI) | Mortality aHR (95% CI) |
|---|---|---|---|---|
| LAA morphology | 2.90 (1.02–8.23) | 2.33 (0.93–5.84) | 1.60 (0.70–3.64) | 1.66 (0.54–5.11) |
| $CHA_2DS_2$-VASc score | 1.37 (0.99–1.91) | 1.37 (1.003–1.86) | 1.36 (1.02–1.81) | 1.15 (0.80–1.64) |
| Number of PES | | | | |
| 0 | Ref | Ref | Ref | Ref |
| 1 | 3.28 (0.42–25.55) | 3.62 (0.47–27.86) | 1.85 (0.42–8.20) | 1.55 (0.18–13.24) |
| ≥2 | 2.26 (0.24–21.16) | 2.45 (0.27–22.13) | 1.36 (0.26–7.19) | 3.10 (0.35–27.50) |

Outcomes are presented as the hazard ratio (95% confidence interval) for values of non-chicken wing compared to chicken wing left atrial appendage morphology.

aHR, adjusted hazard ratio; LAA, left atrial appendage; PES, potential embolic source

Supplementary Table 3. Multivariable Cox proportional hazards regression analysis investigating the association between variables and outcomes (model 3).

| Variables | Embolic stroke recurrence aHR (95% CI) | Ischemic stroke recurrence aHR (95% CI) | Any stroke recurrence aHR (95% CI) | Mortality aHR (95% CI) |
|---|---|---|---|---|
| LAA morphology | 2.95 (1.03–8.43) | 2.31 (0.91–5.83) | 1.58 (0.69–3.63) | 1.44 (0.44–4.71) |
| Hypertension | 1.41 (0.48–4.10) | 1.35 (0.50–3.66) | 1.31 (0.52–3.33) | 1.02 (0.29–3.63) |
| Diabetes mellitus | 0.91 (0.29–2.89) | 1.46 (0.54–3.95) | 1.55 (0.61–3.93) | 1.43 (0.39–5.25) |
| Hyperlipidemia | 1.25 (0.47–3.32) | 1.30 (0.52–3.23) | 1.24 (0.53–2.92) | 0.93 (0.29–2.95) |
| Supraventricular tachycardia or subclinical AF | 1.44 (0.51–4.05) | 1.19 (0.44–3.22) | 1.00 (0.38–2.64) | 2.76 (0.89-8.62) |

Outcomes are presented as the hazard ratio (95% confidence interval) for values of non-chicken wing compared to chicken wing left atrial appendage morphology.

aHR, adjusted hazard ratio; LAA, left atrial appendage; AF, atrial fibrillation
