# Supplementary material for: Impact of Left Atrial Appendage Morphology on Recurrence in Embolic Stroke of Undetermined Source and Atrial Cardiopathy
Source: Front Neurol. 2021 Jun 22;12:679320. doi: 10.3389/fneur.2021.679320 (PMC8258144; doi:10.3389/fneur.2021.679320)
Supplement: Supplementary file 2 [file Image_1.PDF]

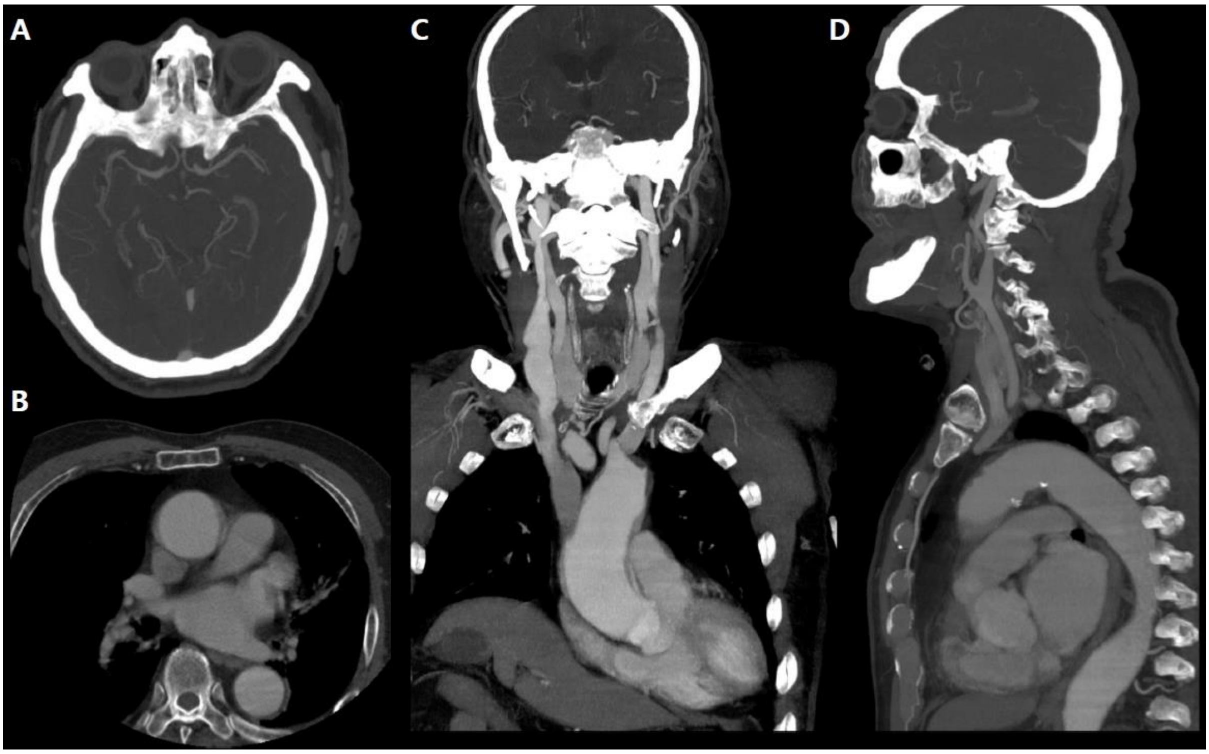

Supplementary Figure. The CCTA protocol involved the following steps: contrast-enhanced multidetector CT was performed using a Revolution Evo Scanner (General Electric, Milwaukee, WI) with iomeprol (Iomeron 400: Bracco Diagnostics, Milan, Italy) injected into the great saphenous vein using an 18-gauge catheter. Nonelectrocardiogram-gated acquisition from the lower limit of the cardiac apex to the intracranial arteries was performed with the following parameters: feet-to-head direction, section thickness (1.25 mm), pitch (1.0), tube voltage (100 kV), amperage (150 mAs per section), reconstruction filter B, and bolus tracker set on the aortic arch (attenuation threshold, 200 HU). Then, 50 mL iomeprol followed by 60 mL saline solution were injected at 4 mL/s for a total injected contrast material volume of 120 mL. An illustrative case is presented here. MIP image of axial CTA of brain **(A)**, axial image of chest section **(B)**, coronal and sagittal CTA of neck and chest section **(C-D)**. CCTA, cerebral computed tomography angiography; MIP, maximum intensity projection; CTA, computed tomography angiography.
